# Are polymorphisms in MTRR A66G and MTHFR C677T genes associated with congenital heart diseases in Iranian population? 

**DOI:** 10.22088/cjim.8.2.83

**Published:** 2017

**Authors:** Noormohammad Noori, Ebrahim Miri-Moghaddam, Asieh Dejkam, Yasman Garmie, Ali Bazi

**Affiliations:** 1 Department of Pediatric Cardiology, Children and Adolescents Health Research Center, Zahedan University of Medical Sciences, Zahedan, Iran.; 2 Genetics of Non-Communicable Disease Research Center, Zahedan University of Medical Sciences, Zahedan, Iran.; 3 CardioVascular Diseases Research Center, Birjand University of Medical Sciences, Birjand, Iran; 4 Cellular and Molecular Research Center, Zahedan University of Medical Sciences, Zahedan, Iran.; 5 Clinical Research Development Unit, Amir-Al-Momenin Hospital, Zabol University of Medical Sciences, Zabol, Iran

**Keywords:** 5, 10-methyleneterahydrofolate reductase (MTHFR), Methionine synthase reductase (MTRR), congenital heart defects (CHDs), Ventricular septal defect (VSD), Tetralogy of fallot (TOF)

## Abstract

**Background:**

The 5, 10-methyleneterahydrofolate reductase (MTHFR) and methionine synthase reductase (MTRR) are two essential enzymes involved in folate metabolism. The relationship between genetic polymorphisms and congenital heart defects (CHDs) is inconsistent. Our aim was to investigate the association between two well-known polymorphisms of MTHFR and MTRR genes, C677T and A66G, respectively, and CHDs in Iranian patients.

**Methods:**

We enrolled 74 patients with ventricular septal defect (VSD) and 79 with tetralogy of fallot (TOF) along with 147 healthy controls. C677T and A66G polymorphisms were detected using tetra-primer ARMS (amplification refractory mutation system) PCR.

**Results:**

Individuals carrying homozygote mutant (TT) genotype of C677T polymorphism represented the highest risk for CHDs (OR=7.3, 95% CI: 0.8-61, P=0.06). Also, significantly increased risk of VSD was observed in individuals with TT genotype (OR=10, 95% CI: 1-92.2, P=0.04). However, the frequency for variant allele (T) of C677T polymorphism was not statistically different between cases and controls (16.3% and 20.9%, respectively). For A66G polymorphism, we found that AG and GG genotypes had higher frequencies in the patients (48.4% and 21.6% respectively) than controls (42.9% and 15.6%, respectively). In line with this, combined AG+GG genotype represented with significantly elevated risk of CHDs (OR=1.6; 95% CI: 1-2.6, P=0.03). AG+GG combination was also identified as a risk factor for TOF (OR=1.8, 95% CI: 1-3.3, P=0.04).

**Conclusion:**

We demonstrated that C677T polymorphism of MTHFR gene was significantly associated with VSD in our patients. Our study also suggested that A66G polymorphism of MTRR gene may contribute to the development of TOF in Iranians.

Congenital heart diseases (CHDs) are among the most common forms of heart abnormalities in children, and are considered as primary causes of premature death worldwide (1, 2). Ventricular septal defects (VSD) and tetralogy of fallot (TOF) represent the most common forms of CHDs in different populations (3). Both genetic and acquired factors are supposed to take part in CHDs pathogenesis; nevertheless, higher risk of CHDs in first-degree relatives highlights the role of genetic etiological context (4, 5). Folic acid is a vital vitamin required to ensure sufficient supplement of one-carbonated (methyl) groups for synthesis and methylation of DNA. 

These roles are ensured through recruitment of folate biological derivatives in enzymatic reactions of thymidine and methionine synthesis (6). In the metabolic process of folate, 5, 10-methyleneterahydrofolate reductase (MTHFR) is the first enzyme catalyzing the conversion of 5,10-methyleneterahydrofolate to 5-methyltetrahydrofolate. Subsequently, 5-methyltetrahydrofolate serves as methyl donor for synthesis of methionine from homocystein by methionine synthase (MTR) (7). In parallel, methionine synthase reductase (MTRR), another essential enzyme in folate metabolism, reduces the cobalamin cofactor of MTR to provide continuous methionine synthesis cycle.

C677T and A66G polymorphisms are two common genetic variants of MTHFR and MTRR genes known to reduce activities of the respective enzymes (3). C677T polymorphism resides in exon 4 of MTHFR gene, and is associated with 30-60% fall in enzyme activity (8). Recent polymorphism has been associated with low level of folate bioavailability for methionine synthesis (9, 10). Likewise, A66G polymorphism of MTRR results in diminished rate of methionine synthesis and elevated homocystein level in plasma (7, 11). In return, smaller methionine pool leads to inadequate production of S-adenosyl methionine (SAM), the main methyl group donor in methyl transfer reactions, including DNA methylation, which causes DNA instability (11). 

In recent decades, the role of genetic polymorphisms in propensity to CHDs has acquired attention (12, 13). In this regard, C677T polymorphism of MTHFR gene has been reported to significantly boost the risk of CHDs in Egyptian (3, 14) Asian (15) and Chinese (16) populations. Interestingly, the presence of C677T polymorphism in mothers has markedly elevated the risk of CHDs in the siblings (6, 17). Similarly, A66G polymorphism of MTRR has also been suggested as a risk factor for CHDs by some studies (18, 19). Moreover, the association of A66G polymorphism with CHDs is less clarified, as some reports failed to demonstrate a significant relationship (20). 

From another point of view, the role of C677T and A66G polymorphisms is less studied in specific subtypes of CHDs.* When considering CHDs as heterogeneous subtypes, the risk association conclusions are uncertain*. Besides, it seems that the influence of these polymorphisms on the risk of CHDs development is modulated depending on the population under study (21). Nevertheless, the number of studies assessing genetic polymorphisms of folate related genes in individual subtypes of CHDs is handful in the literature. To our knowledge, there is only one study investigating the role of C677T and A66G polymorphisms in Iranian VSD patients (11). Therefore, we aimed to assess the impact of C677T polymorphism of MTHFR and A66G polymorphisms of MTRR on the risk of two common CHDs subtypes, VSD and TOF in an Iranian population.

## Methods

**Sample population: **This case-control study was performed on 153 patients diagnosed with CHDs (74 VSD and 79 TOF cases) recruited from Cardiac Care Center of Imam Ali Hospital in Zahedan, Sistan and Balouchistan province, southeast of Iran during 2014-2015. 147 healthy sex-age-and ethnically matched case participants were selected. Diagnosis of CHDs was confirmed using specified diagnostic techniques of echocardiography, cardiac catherization and surgical procedures. The controls also underwent echocardiography to ensure the absence of cardiac conditions. Parents were requested to sign an informed contest, and likewise were interviewed for a family history of heart diseases. Our study was approved by the Ethics Committee of Research Deputy of Zahedan University of Medical Sciences.

**Polymorphism genotyping: **For obtaining DNA, 2 ml blood samples in EDTA anticoagulant were obtained from the participants. Genomic DNA was extracted by a standard protocol exploiting proteinase k enzyme (22). Tetra-ARMS (amplification refractory mutation system) PCR was the method of choice to detect polymorphisms of A66G and C677T. In this method, two pairs of primers (one pair of inner primers and one pair of outer primers) were designed for each polymorphism.

**PCR reaction: **Amplification of A66G polymorphism was carried out using inner primer with sequences of forward; (A allele): (5'–AAG GCC ATC GCA GAA GAA CTA -3') and backward; (G allele): (5'–CAT GTA CCA CAG CTT GCT CAA AC -3') and outer primer with sequences of forward; 5' –ACA TGC CTT GAA GTG ATG AGG A -3' and backward5' –CCC AAC CAA AAT TCT TCA AAG C -3'. For C677T polymorphism. sequences of inner primer were forward, T allele: 5'-AGA AGG TGT CTG CGG GCG T -3' and backward C allele:5'-AAA GCT GCG TGA TGA TGA AAT AGG -3' as well as outer primes which had sequence of forward; 5'-GCT GTT GGA AGG TGC AAG ATC A -3' and backward; 5'-GAG TGG GGT GGA GGG AGC TTA T -3'. Wilde-type (A) and variant (G) alleles of A66G polymorphism were identified by sizes of 140 bp and 102 bp, respectively. As well, product sizes were 177 bp and 230 bp for T and C alleles, respectively. PCR amplification was performed on 1 µl of 100 ng/ml genomic DNA template admixed with 1.5 µl of each inner and 0.9 µl of each outer primer. Taq premix 2X (GenetBio, Korea) as well as 5 µl dd H_2_O was also included into reaction tube. PCR was performed as 30 cycles of denaturation, annealing and amplification phases using appropriate temperature profiles (Eppendorf, Germany thermal cycler).Briefly, a common 5- minute in 95℃ following with 30 cycles of 95℃ (denaturation), 61℃ for C677T and 56℃ for A66G polymorphism (annealing) and 72℃ (extension) each for 30 seconds, as well as a final 5-minute period of extension phase was considered.

**Statistical analysis: **SPSS statistics Version 19 was used to conduct the analysis. In appropriate place, statistical tests such as Chi-square, t student –independent and logistic regression were performed to detect significant associations, differences and correlations.

## Results

From 153 CHDs patients (74 VSD and 79 TOF cases) 84 (54.9%) were males (including 43 (58.1%) in VSD and 41 (51.9%) in TOF). In comparison, respective gender ratios were 70 (47.6%) and 77 (52.4%) for males and females in controls (P>0.05). There was no significant difference between the mean age of controls (4.9±3.9) and patients with TOF (4.2±3.8); though the patients with VSD had significantly lower mean age (3.4±3.2, P=0.006). 

**Figure 1:**
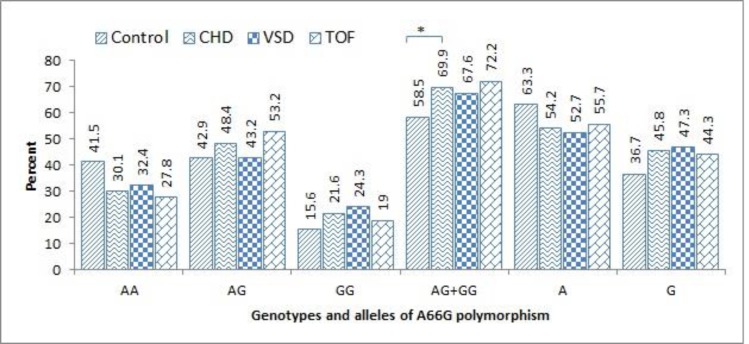
Frequencies of genotypes and alleles of A66G polymorphism of MTRR gene in the controls and the patients with CHDs. Figure indicates percentages of each genotype and allele in total population of the patients, as well as in separate subgroups of the patients with VSD and TOF

**Figure 2:**
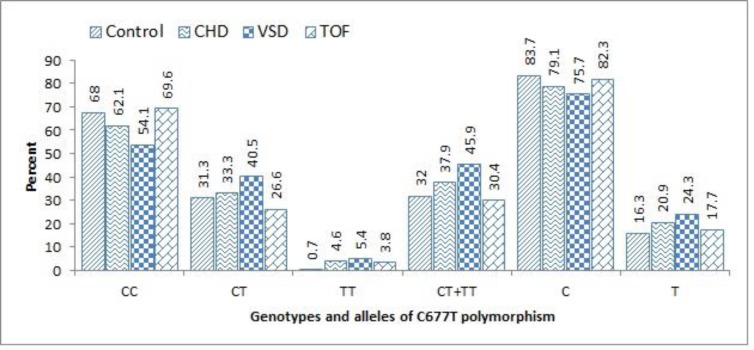
Frequencies of genotypes and alleles of C677T polymorphism of MTHFR gene among patients with two common types of CHDs (VSD and TOF), and its comparison with control group

Frequencies of different genotypes of A66G and C677T polymorphisms in controls, CHDs, as well as separate VSD and TOF patients have been represented in figures 1 and 2, respectively. Heterozygote (AG) genotype of A66G polymorphism was the most identified (48.4%) in the patients. Although the difference for AG distribution was not statistically significant between case and control group, when considering combined AG+GG genotypes, patients had significantly higher occurrence (69.9%) compared to controls (58.5%, P=0.03). 

For both groups, wild-type allele (A) represented with higher frequency than variant; nevertheless, mutant allele had higher frequency in cases (45.8%) than controls (36.7%). Regarding C677T polymorphism, the most noticeable feature was significantly the higher frequency of homozygote mutant genotype (TT) in patients (4.6%) than controls (0.7%) (OR=7.3, 95% CI: 0.8-61, P=0.06). Similar to the results obtained for A66G polymorphism, wild type allele of C677T polymorphism was also detected in the majority of participants in both the case (79.1%) and controls (83.7%).

Results of logistic regression analysis for determining the overall risk of CHDs, and also the risk of individual VSD or TOF diseases have been shown in table 1. Genotypes containing mutant allele of A66G polymorphism, heterozygote (AG) and homozygote (GG), have been associated with elevated risk of both the VSD (OR=1.9, P=0.08) and TOF (OR=1.8, P=0.05). When treating homozygote wild type genotype (AA) as reference, combinations of heterozygote and mutant homozygote (AG+GG) of A66G polymorphism significantly increased the risk of TOF (OR=1.8, 95% CI:1-3.3, P=0.04). Considering C677T polymorphism, heterozygote (CT) genotype was detected in higher rate in VSD than TOF patients (40.5% versus 26.6%, P=0.06). Notably, mutant homozygote genotype of C677T polymorphism was significantly associated with the risk of VSD (OR=10, P=0.04). 

**Table 1:** Logistic regression analysis for estimating the overall risk of CHDs, and risk of VSD or TOF in the presence of different genotypes and alleles of A66G and C677T polymorphisms

**Polymorphisms**		**CHDs (n=153)**			**VSD (n=74)**			**TOF(n=79)**		
		n(%)	OR(95% CI)	P	n(%)	OR (95% CI)	P	n(%)	OR(95% CI)	P
A66G	AA	46(30.1)	Ref	-	24(32.4)	Ref	-	22(27.8)	Ref	-
	AG	74(48.4)	1.5(0.9-2.5)	0.08	32(43.2)	1.2(0.5-2.4)	0.4	42(53.2)	1.8(0.9-3.4)	0.05
	GG	33(21.6)	1.9(0.9-3.6)	0.05	18(24.3)	1.9(0.9-4.3)	0.08	15(19)	1.8(0.8-4)	0.1
	AG+GG	107(69.9)	1.6(1-2.6)	0.03	50(67.6)	1.4(0.8-2.6)	0.1	57(72.2)	1.8(1-3.3)	0.04
	A		Ref	-	52.7	Ref	-	55.7	Ref	-
	G		1.4(0.9-2.3)	0.1	47.3	1.5(0.8-2.7)	0.1	44.3	1.3(0.7-2.3)	0.2
C677T	CC	95(62.1)	Ref		40(54.1)	Ref	-	55(69.6)	Ref	-
	CT	51(33.3)	1.1(0.7-1.9)	0.5	30(40.5)	1.6(0.9-2.9)	0.1	21(26.6)	0.8(0.4-1.5)	0.5
	TT	7(4.6)	7.3(0.8-61)	0.06	4(5.4)	10(1-92.2)	0.04	3(3.8)	5.4(0.5-53.7)	0.1
	CT+TT	58(37.9)	1.2(0.8-2)	0.2	34(45.9)	1.8(1-3.2)	0.04	24(30.4)	0.9(0.5-1.6)	0.8
	C allele		Ref		75.7	Ref	-	82.3	Ref	-
	T allele		1.5(0.7-2.4)	0.3	24.3	1.6(0.8-2.3)	0.1	17.7	1.1(0.2-2.2)	0.7

CHD: congenital heart disease

VSD: ventricular septal defect

TOF: tetralogy of fallot

Most common recognized haplotypes in controls were A66G:C677T; AA:CC (32%), A66G:C677T; AG:CT (23.8%) and A66G:C677T; AG:CC (20.4%). In CHDs, still, the most identified haplotype was A66G:C677T; AG:CC (30.1%). Interestingly, haplotypes with a mutant allele of either A66G or C677T polymorphisms had significantly higher risk of CHDs. Specifically, A66G:C677T; AA:CT haplotype (OR=4.1, 95% CI: 1.7-9.8, P=0.001), A66G:C677T; AG:CC (OR=3.4, 95%CI: 1.7-6.8,P=0.0001), A66G:C677T; GG:CC (OR=2.5, 95% CI: 1-5.8, P=0.03) and A66G:C677T; GG:CT (OR=5.2, 95% CI: 1.7-15.4, P=0.003) were haplotypes most strongly associated with risk of CHDs. Risk association of different haplotypes in specific subtypes of VSD and TOF have been presented in table 2.

**Table 2:** Haplotypes recognized in healthy controls compared to ventricular septal defect (VSD) and tetralogy of fallot (TOF) patients.

**Parameter**		**Control (n=147)** **n (%)**	**VSD (n=74)**			**TOF(n=79)**		
			**n (%)**	**OR (95% CI)**	**P**	**n (%)**	**OR (95% CI)**	**P**
**Haplotypes**	AA+CC	47(32)	9(12.2)	Ref	-	12(15.2)	Ref	-
	AA+CT	12(8.2)	12(16.2)	5.2(1.7-15.2)	0.003	10(12.7)	3.2(1.1-9.3)	0.02
	AA+TT	1(0.7)	3(4.1)	15.6(1.4-168)	0.02	0(0)	-	-
	AG+CC	30(20.4)	20(27)	3.4(1.4-8.6)	0.007	26(32.9)	3.3(1.4-7.7)	0.004
	AG+CT	35(23.8)	12(16.2)	1.7(0.6-4.7)	0.2	13(16.5)	1.4(0.5-3.5)	0.4
	AG+TT	0(0)	0(0)	-	-	3(3.8)	-	-
	GG+CC	16(10.9)	6(8.1)	1.9(0.6-6.3)	0.2	12(15.2)	2.9(1.1-7.8)	0.03
	GG+CT	6(4.1)	11(14.9)	9.5(2.8-32.5)	0.0001	3(3.8)	1.9(0.4-8.9)	0.3
	GG+TT	0(0)	1(1.4)	-	-	0(0)	1.8(1.8-1.8)	-

VSD: ventricular septal defect

TOF: tetralogy of fallot

## Discussion

The main purpose of the current study was to assess the impact of two common polymorphisms in folate metabolic- related genes, C677T polymorphism of MTHFR and A66G polymorphism of MTRR, on the risk of VSD and TOF in an Iranian population. We found that haplotypes bearing mutant alleles of either polymorphism significantly had higher risk of cardiac disorders. In fact, the highest risk of CHDs was observed in subjects carrying A66G:C677T; GG: CT haplotype (OR=5.2, P=0.003). We found no study on CHDs to compare such effects with our results. Nevertheless, one possible mechanism for this observation can be the cumulative effect of these polymorphisms in raising homocystein level of plasma. (23). 

 It has been described that synergistic behavior of both variant alleles of C677T and A66G polymorphisms with alleles of other genes related to folate metabolism, such as MTR, glutamate carboxypetidase II (GCP II) and methionine synthase (MS), can influence the risk of coronary artery disease (CAD) in different populations (24-26). In line with these observations, the significant elevated risk of CAD was also reported in the copresence of both C677T polymorphism of MTHFR and A66G polymorphism of MTRR (27). Although hyperhomocystenemia has been mentioned as the main intruder associated with increased risk of cardiac abnormalities in the setting of presence of multiple polymorphisms, the role of environmental factors as effectors including consumption of vit B12 and folate supplements should also be considered (25, 27).

When treating CHDs as a unit entity, homozygote mutant genotype of C677T polymorphism enhanced the risk of disease (OR= 7.3, P=0.06). 

 In regression analysis for individuals subtypes, C677T polymorphism of MTHFR seemed to be significantly associated with the risk of VSD (OR=10, 95% CI: 1-92.2, P=0.04). In comparison to our findings, homozygote state of mutant allele of C677T polymorphism was reported to significantly accentuate the risk of CHDs in Egyptian patients (OR=2.6, p=0.004) (3). In accordance are also the studies of Zhu et al (16), and El-Abd et al. (28) who reported the significant risk of TT genotype of C677T polymorphism for CHDs. In the recent study, TT genotype has been specifically associated with the risk of VSD (OR=5.2, P=0.02) (28). In contrast, no association was found between C677T polymorphism and CHDs in the studies by Pishva et al. on Iranian patients (11) and Kotby A et al. on Egyptian children (29). Nonetheless, the representation of no individuals with TT genotype is probably the explanation for the mentioned result in the study of Pishva et al. (11). In comparison to other genotypes of C677T polymorphism, TT genotype also represented as most strongly associated with the risk of TOF (OR=5.4, P=0.1). Similarly, this genotype markedly increased the risk of TOF in Chinese patients (OR=1.6, P=0.04) (30). Furthermore, Portuguese researchers have reported an increased risk of TOF with the inheritance of double dose of T allele of C677T polymorphism (OR=4.8) (31). Collectively, these results highlight a potential role for C677T polymorphism in the pathogenesis of both VSD and TOF. C677T polymorphism is known to decrease folate level of serum, as heterozygote and variant homozygote genotypes of this polymorphism are characterized with respectively 30% and 70% reduction in enzyme activity (3). Low level of folate bioavailability is related to higher homocystein level of plasma, and in turn increased the risk of cardiovascular diseases (32). More importantly, inadequate methionine source can disrupt DNA methylation process, and therefore influence cellular differentiation (6, 13). Conclusively, the observed risk of C677T polymorphism of MTHFR gene for CHDs may be related to abnormal vascular tone and nucleic acid biology. 

Our results also demonstrated a role for A66G polymorphism in the pathogenesis CHDs. In general, both heterozygote and mutant homozygote genotypes of this polymorphism were closely related to CHDs (OR=1.5 for AG, P=0.08 and OR= 1.9 for GG, P=0.05). In individual states, as regards AA genotype as a reference, combinations of AG+GG genotype showed highlighted risk of TOF(OR=1.8, 95% CI: 1-3.3, P=0.04). Additionally, homozygote mutant genotype (GG) also rendered as a risk factor for VSD (OR=1.9, 95% CI: 0.9-4.3, P=0.08). Despite this, the association of G allele with neither VSD nor TOF was not statistically significant which may relatively root in a study with low power. 

In comparison, A66G polymorphism of MTRR was revealed as a risk factor for CHDs specially in the Asian population(13). Likewise, the polymorphism was reportedly associated with CHDS by other authors (7, 19). In contrast, there are also reports that have either described no association between A66G polymorphism and the CHDs (11, 20, 33), or noted a protective role for A66G polymorphism against CHDs (34). However, the recent study contained a highly heterogeneous population of patients constituted of ten subgroups of CHDs which may affect the association analysis (34). 

In addition to heterogeneous patient populations, conflicting data obtained for A66G polymorphism may represent the impact of other genetic or environmental determinants. In particular, the nutritional status of cobalamine may modulate the effect of MTRR polymorphisms on the risk of CHDs. Accordingly, a continuous supply of cobalamin is crucial for methyltransferase activity during MTR (18). Therefore, consumption of multivitamins during childbearing period may influence the risk of CHDs. Due to limitation in the availability of such regimens by mothers, we were unable to include such parameter in our study. There were also studies suggesting ethnicity as a determining factor influencing the impact of A66G polymorphism on the risk of CHDs (13, 35). From the other point of view, the risk of specific subtypes of CHDs may also be differentially modified by polymorphisms residing in genes involved in folate metabolism (34). In conclusion, more studies are required to establish a role of A66G polymorphism in CHDs. 

In Conclusion, overall, our study showed the role of C677T polymorphism in the pathogenic process of VSD and A66G polymorphism in TOF. Genetic polymorphism involved in folate metabolism seem to influence the risk of CHDs development. Specifically, our results indicate that the role of particular genes and consequently enzymatic reactions may contribute in developing certain cardiac pathologies. Yet, more studies are encouraged to elucidate this.
